# Auxiliary occlusal devices for IO scanning in a complete digital workflow of implant-supported crowns: a randomized controlled trial

**DOI:** 10.1186/s12903-024-03986-4

**Published:** 2024-03-22

**Authors:** Shuxin Ren, Xi Jiang, Ping Di

**Affiliations:** Department of Oral Implantology, Peking University School and Hospital of Stomatology, 22 Zhongguancun South Avenue, Haidian District, Beijing, 10081 PR China

**Keywords:** An auxiliary device, Multiple implants, Digital impression, Crown accuracy

## Abstract

**Objectives:**

To compare the crown accuracy and time efficiency of a complete digital workflow, utilizing an auxiliary occlusal device and IO scanning, with a conventional workflow, for multiple implant-supported single crowns.

**Materials and methods:**

24 patients with two adjacent posterior implants were included. 12 patients were randomly assigned to digital workflow group, involving intra-oral scanning with an auxiliary occlusal device and manufacture of customized abutments and zirconia single crowns (test group). The other 12 were assigned to the conventional workflow (control group), involving conventional impression and CAD-CAM crowns based on stone casts. Crown scanning was done before and after clinical adjustment using an intraoral scanner. Two 3D digital models were overlapped to assess dimension changes. Chair-side and laboratory times for the entire workflow were recorded and a linear mixed model and Independent-sample t tests were used for the statistical analysis.

**Results:**

The maximum occlusal deviation was 279.67 ± 112.17 μm and 479.59 ± 203.63 μm in the test and control group, respectively (*p* < 0.001). The sizes of the occlusion adjustment areas were 12.12 ± 10.51 mm^2^ and 25.12 ± 14.14 mm^2^ in the test and control groups, respectively (*p* = 0.013). The mean laboratory time was 46.08 ± 5.45 and 105.92 ± 6.10 min in the test and control groups, respectively (*p* < 0.001).The proximal contact adjustment and mean chair-side time showed no statistically significant difference between two groups.

**Conclusions:**

A digital workflow for two implants-supported single crowns using an auxiliary device required fewer occlusal crown adjustments, and less laboratory time compared to conventional workflow.

**Clinical Relevance:**

The use of auxiliary occlusal devices in IOS enhances the accuracy of virtual maxillomandibular relationship in extended edentulous spans. Consequently, employing a digital workflow for multiple implants-supported crowns using IO scanning and an auxiliary occlusal device proves to be a feasible, accurate and efficient approach.

## Introduction

When using intra-oral scanning (IOS) for partially edentulous arches, it is challenging to obtain an accurate virtual relationship between the upper and lower jaws. IOS captures buccal images of the maxillary and mandibular teeth in the proposed position as virtual interocclusal records (VIRs) [1, 2]. Digital maxillary and mandibular casts obtained from intra-oral scanning (IOS) can be repositioned in CAD software using VIRs and best-alignment algorithm, which orients various 3D files by pairing corresponding data [3]. For a single missing posterior tooth, VIRs can achieve a high level of accuracy comparable to that of the dentate condition. This contributes to the apparent occlusal accuracy of crowns fabricated with the digital workflow, as demonstrated in previous studies [4–6]. However, when more than one tooth is absent, an expanded edentulous span with large amounts of mobile tissue provides few landmarks in VIRs, leading to decreased accuracy. Several laboratory experiments revealed that VIRs have insufficient accuracy for extended edentulous spans with three or more missing teeth, resulting in compromised maxillomandibular alignment and poor occlusal accuracy of crowns [1, 7, 8]. Consequently, limited clinical trials have reported the use of full digital workflows for partial edentulous spans with more than one missing tooth, with some studies [9, 10] not evaluating restoration accuracy or occlusion and others [11, 12] utilizing interim prostheses for maxillomandibular relationship registration, which adds to the overall cost and is not a standard step in restoring partial edentulous spans. Therefore, obtaining accurate virtual maxillomandibular relationship solely through IOS remains a technical challenge for restoring multiple implant-supported crowns in a digital workflow.

In recent years, auxiliary devices have been introduced to the intra-oral scanning process in order to improve the accuracy of digital impressions for full-arch implant rehabilitation [13–16]. The accuracy of digital impressions for complete edentulous arches has been limited by the long distances of soft tissue between implant scanbodies, which are difficult to identify using intra-oral scanners (IOS). However, by adding artificial landmarks placed on edentulous areas and between implant scanbodies, more geometry with a differentiating radius of curvature can be captured by the scanner [13–16]. This distinct geometry facilitates the stitching process and significantly improves the accuracy of digital impressions. Similarly, lack of geometry on an edentulous area also compromises the accuracy of VIRs, but this problem can be overcome by using auxiliary devices [17].

Jin et al. [17] reported the use of healing abutments on the implants significantly improves the VIRs of partial edentulous area in laboratory setting. However, no clinical study was available to explore improving the accuracy of VIRs with the help of auxiliary devices and facilitate their use in restoring multiple implant-supported crowns using a digital workflow.

The objective of this randomized controlled trial (RCT) was to compare the crown accuracy and time efficiency of a complete digital workflow, utilizing an auxiliary occlusal device, with a conventional workflow, for multiple implant-supported single crowns. The null hypothesis stated that there would be no significant difference in crown accuracy and clinical and laboratory times between the complete digital and conventional workflows.

## Materials and methods

The study was a single-center, unblinded, randomized controlled trial of two parallel groups. Participants were assigned to either the complete digital workflow or conventional segmental workflow (allocation ratio 1:1). The study was independently reviewed and approved by the Institutional Review Board of Peking University School and Hospital of Stomatology ( (Ethical approval No.: PKUSSIRB-201,950,165) and registered in a Clinical Trial Registry (ChiCTR No.: ChiCTR2200064819, 19/10/2022). The Consolidated Standards of Reporting Trials (CONSORT) guidelines were used as a framework for this study.

All participants were informed about the study protocol and written consent was obtained. The inclusion criteria consisted of individuals (age ≥ 18 years) requiring two adjacent implants (Camlog Screw Line; Camlog Biotechnologies GmbH, Switzerland) on premolar and molar region with sufficient prosthetic space (vertical height ≥ 5 mm, mesiodistal distance ≥ 12 mm). The exclusion criteria included individuals with severe malocclusion, local or systemic contraindications for implant therapy, adjacent or antagonistic teeth with Class I–III mobility.

**Fig. 1:**
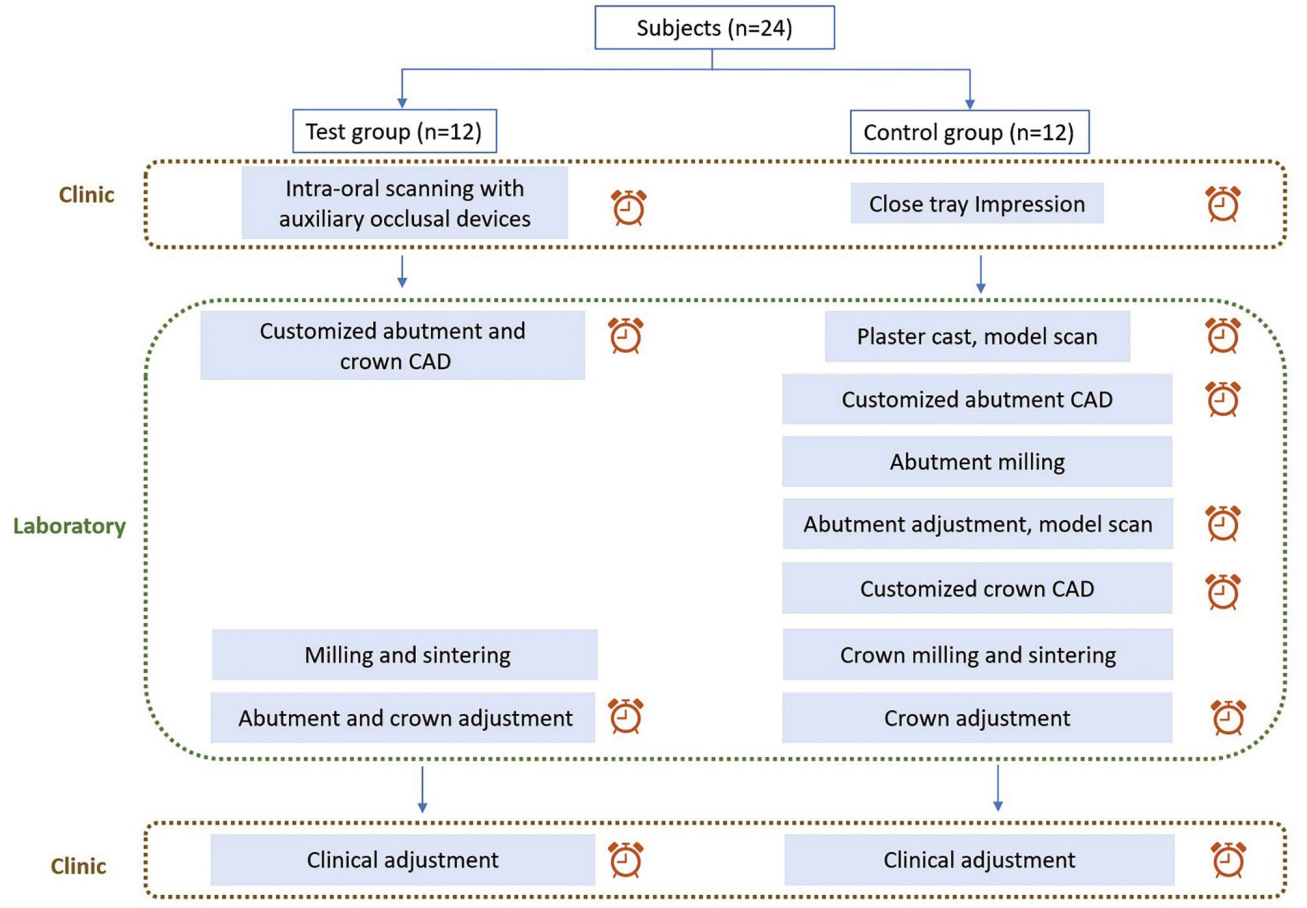
The flow-chart of the study

Twenty-four patients with two posterior implants, placed 4 months previously and ready for impressions, were enrolled and randomly allocated to two groups: the test group underwent intraoral scanning (IOS) with an auxiliary device, model-free computer-aided design/computer-aided manufacturing (CAD/CAM), and crown delivery, while the control group received conventional impressions, stone model-based CAD/CAM, and crown delivery. The process of the study was shown in the flow-chart (Fig. 1).

Randomization was carried out using computer software. An independent researcher not involved in the study placed the computer-generated group numbers into sequentially numbered, opaque, sealed envelopes. The envelopes were opened by the principal investigator in sequential order as participants meeting the inclusion criteria signed the consent form. The dental technician, responsible for crown manufacturing and scanning before and after clinical adjustments, as well as the researcher conducting 3D model measurements, remained blinded to the study design. However, the clinician involved in impression-making and crown deliveries was aware of the groups due to the inherent nature of the study design. The study methods remained unchanged throughout the trial.

**Fig. 2:**
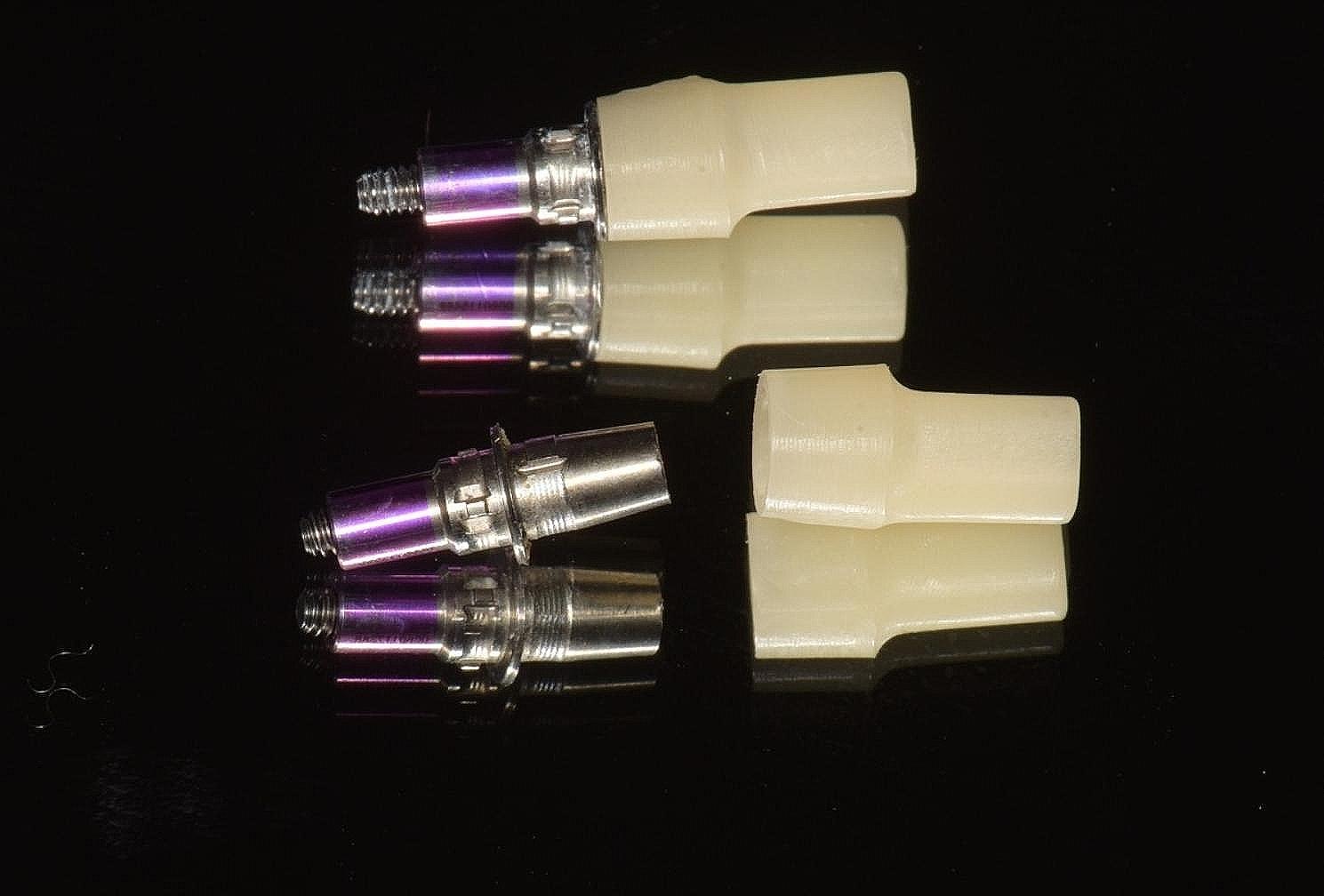
Auxiliary occlusal devices comprised of prefabricated abutments and disposable composite cylinders

**Fig. 3:**
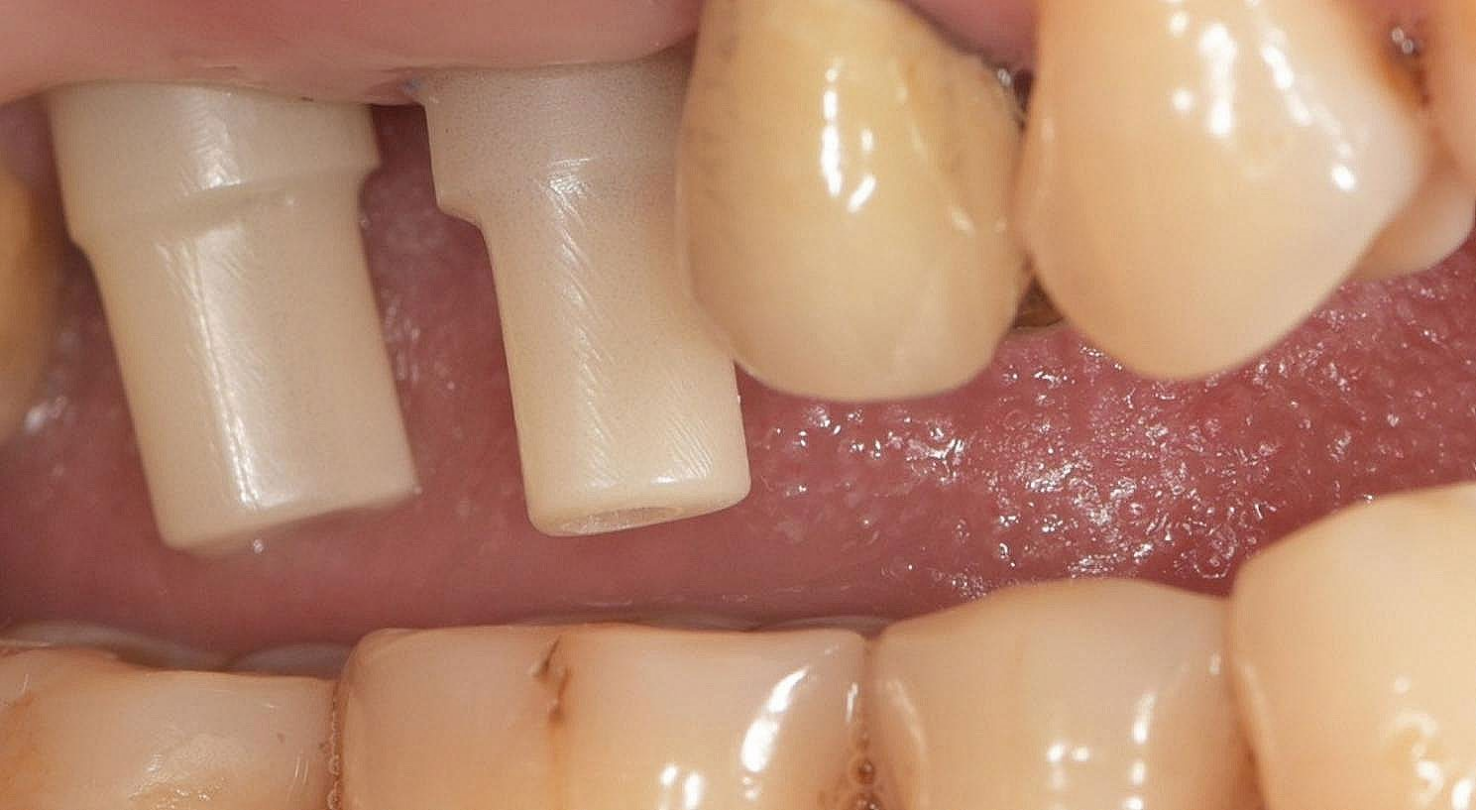
Auxiliary occlusal devices installed to implants

**Fig. 4:**
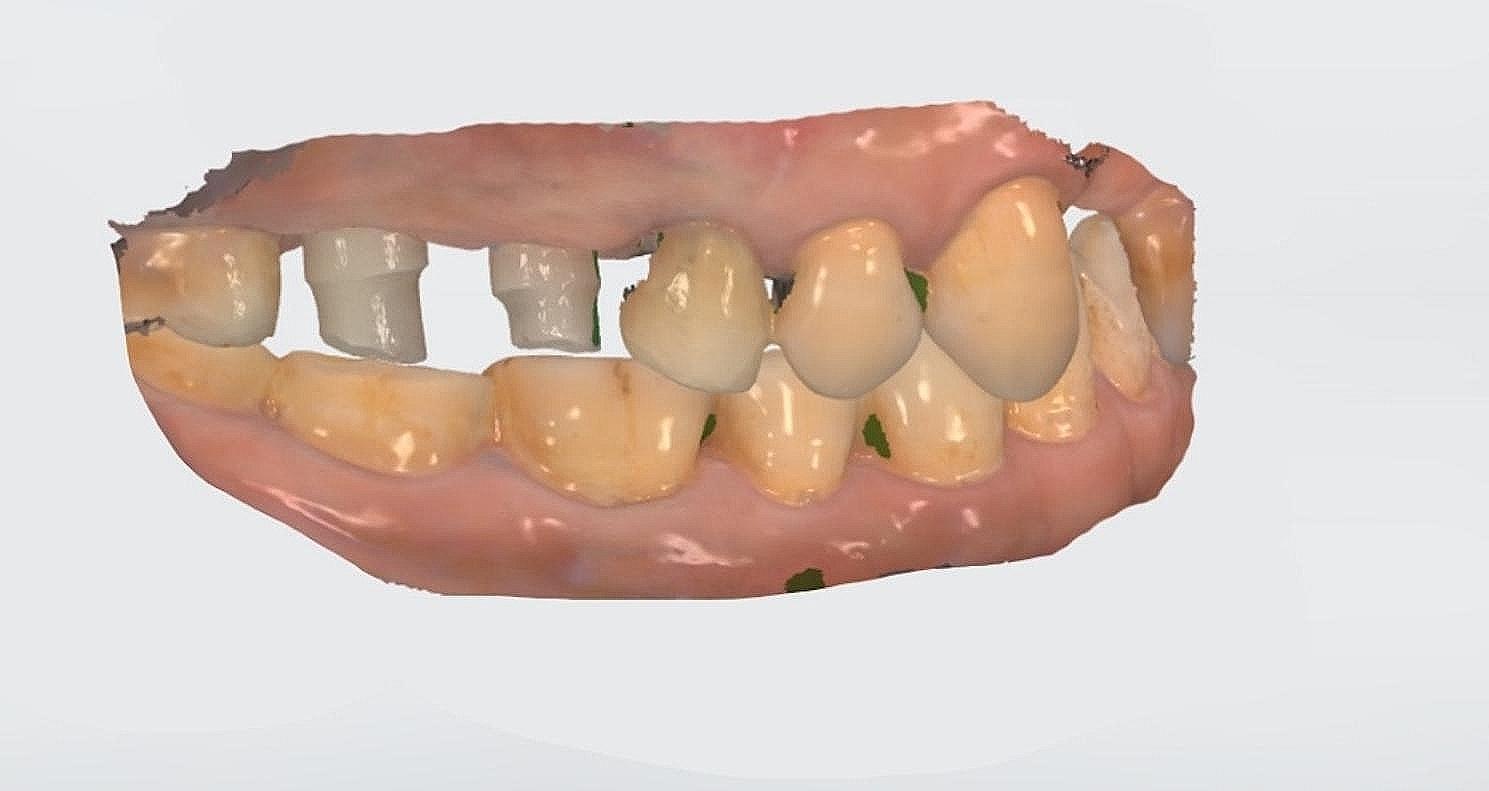
Occlusal ends of auxiliary devices adjusted to achieve occlusion

**Fig. 5:**
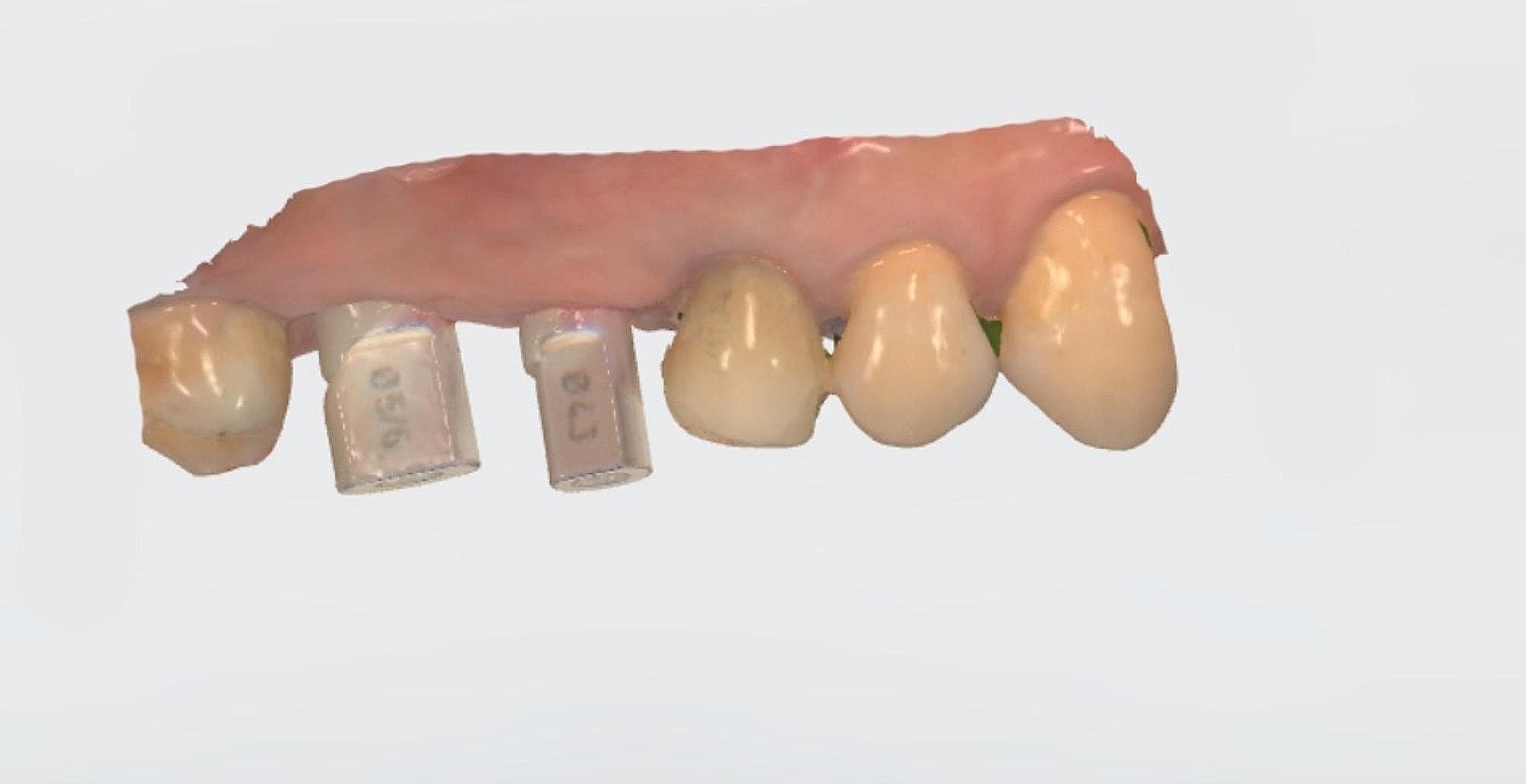
IOS with implant scanbodies

**Fig. 6:**
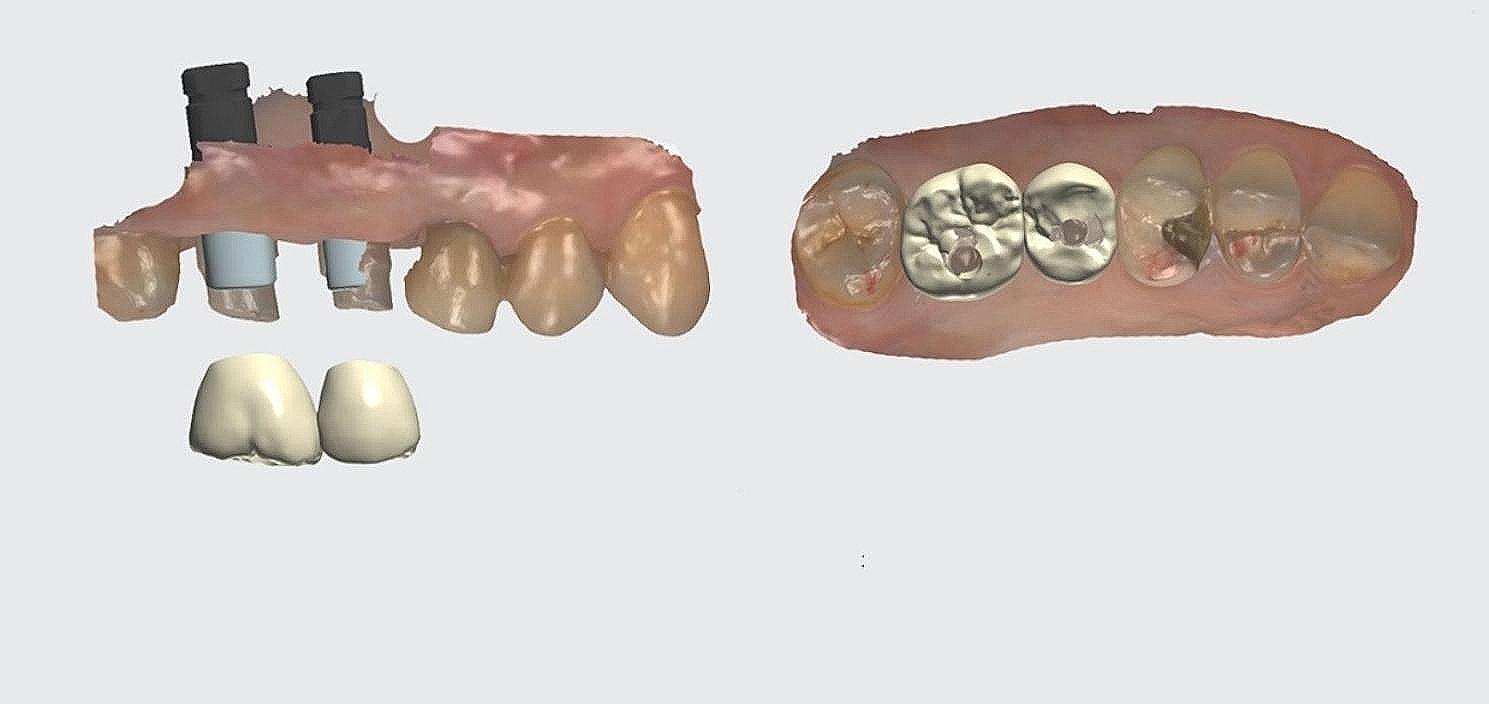
Customized abutments and crowns in CAD

The study involved the installation of auxiliary occlusal devices to implants in the test group. These devices comprise prefabricated titanium abutments and disposable PMMA cylinders that are attached with friction (Fig. 2). The disposable cylinder was designed as a cylinder measuring 15 mm in height and 6 mm in diameter on the virtual Ti-base in CAD software (Dental Designer; 3Shape). The software automatically generated the connection part of the cylinder. Subsequently, the cylinder file was exported and transmitted for manufacturing using proprietary PPMA disk (Leongy, China). Two auxiliary occlusal devices were installed to two implants before impression (Fig. 3). The occlusal ends of composite cylinders were adjusted intraorally until maximum intercuspation position was obtained. Digital impressions of the maxillary and mandibular quadrants, as well as virtual implant restorations (VIRs), were taken using an intra-oral scanner (TRIOS Color; 3Shape, Copenhagen, Denmark) in a controlled environment with a temperature maintained at 20–25ºC. (Fig. 4) Next, the scanbodies (Camlog Biotechnologies, Switzerland) were swapped with the auxiliary occlusal devices, and the edentulous span with scanbodies was rescanned (Fig. 5). The scanning process was conducted solely under the ceiling light, without chair light or natural light sources [18, 19]. The digital models were realigned using the auto-alignment function in the IOS, and the resulting impressions were imported into CAD software (Dental Designer; 3Shape) for further processing. The virtual crowns were designed based on the implants, and then split into customized abutments and crowns, as per the parameters described in a previous study [4]. (Fig. 6) The abutment design file (.STL) was sent to a milling center, where a 5-axis milling machine (Organical Multi 5X; Organical CAD/CAM GmbH, Berlin, Germany) was used to manufacture the titanium abutments. Meanwhile, the crown design file (.STL) was sent to another milling machine (Organical Multi & Changer 20,Organical CAD/CAM GmbH) to produce the crown using proprietary zirconia (Organic Zircon, Organical CAD/CAM GmbH). After milling and sintering, an experienced technician polished and refined the abutments and crowns to improve the marginal fit. No 3D printed models were fabricated during the whole process since the complete digital workflow is model free.

In the control group, conventional silicone closed-tray full-arch impressions were taken. Silicon bite record (Silagum Putty Fast, DMG,Germany) held by 6 mm height healing abutments was used to transfer maxilla-mandibular relationship. Stone models were made and mounted on an articulator with silicon bite record and mounting stone (Mounting Stone, Whip Mix Corp). To prevent gypsum expansion, rubber bands were employed. Models with scanbodies (Camlog Biotechnologies, Switzerland) and maxilla-mandibular relationship were then digitalized by the laboratory scanner (TRIOS; 3shape). Customized titanium abutments were designed using the Dental Designer software and milled using a 5-axis milling machine (Organical Multi 5X). Following adjustments and refinement of the milled abutments by the same technician, the abutments were positioned on the cast with a torque of 20 Ncm. Master casts were again digitalized, and crowns were designed on the abutment using the same software. Zirconia crowns (Organic Zircon) were fabricated using a 5-axis milling machine (Organical Multi & Changer 20) and refined by the technician to improve the marginal fit. This procedure was reported in our previous study [4]. 

Before any clinical adjustments were made, all crowns underwent chair-side scanning in the same controlled environment using the same intraoral scanner (TRIOS Color; 3Shape) to generate STL files marked as “PRE files.”

**Fig. 7:**
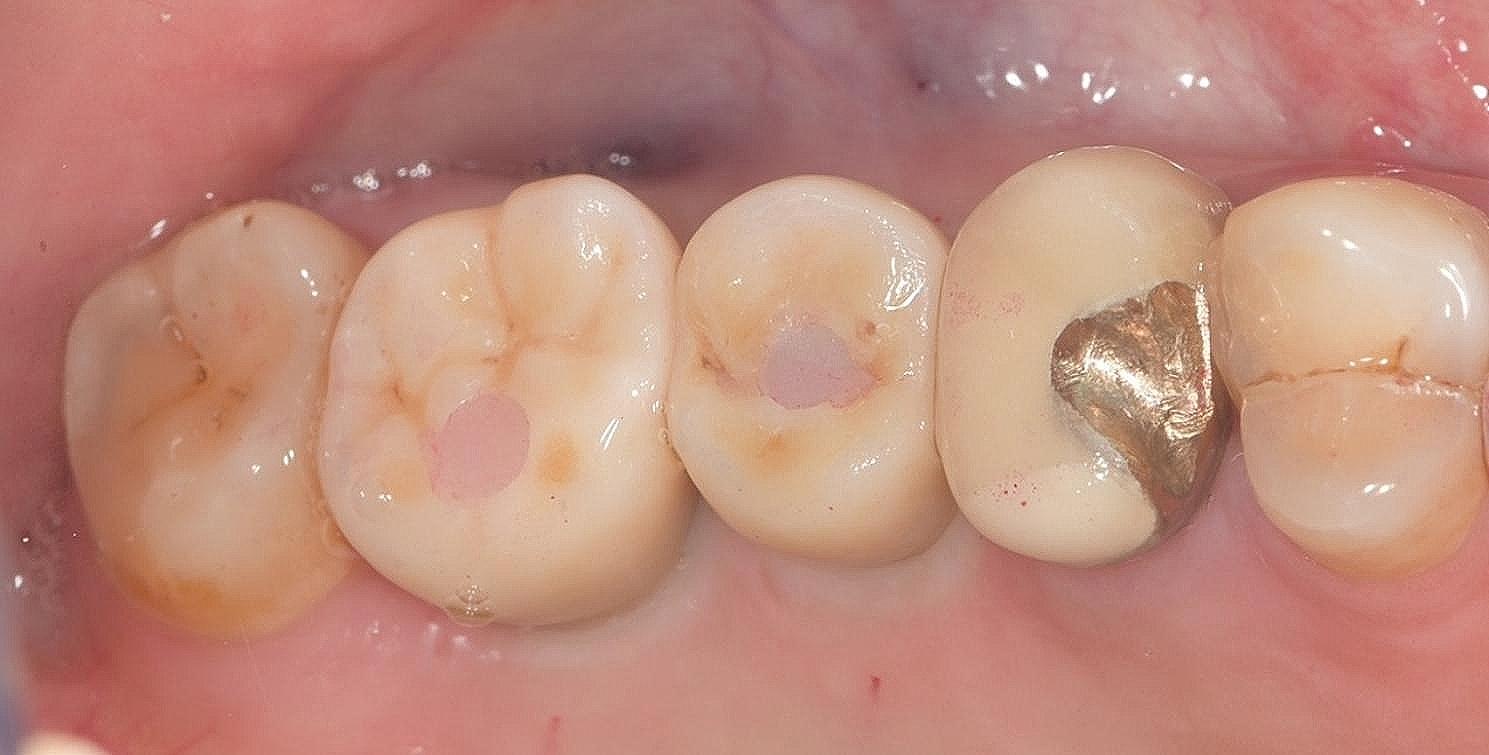
Crown delivery

After screwing the customized abutment into the implant with a torque of 35 Ncm (under local anesthesia if necessary), we assessed the interproximal fit and occlusal contact by inserting a zirconia crown onto the abutment. Interproximal contact was considered favorable if dental floss passed with moderate resistance. Favorable occlusal contact was standardized by light contact without lateral occlusal interference using 40-µm articulating paper (Arti-Fol Shimstock foil; Dr. Jean Bausch GmbH & Co., Köln, Germany) and no contact was present by using 12-µm articulating paper (Arti-Fol Shimstock foil; Dr. Jean Bausch GmbH & Co., Köln, Germany), which could be pulled out with no resistance during light occlusion. Diamond burs and silicone polishers were used to remove premature contact points, and porcelain was added in the laboratory to address missing contact points. After making any necessary adjustments, post-adjustment crowns were scanned using the same scanner to generate new STL files, marked as “POST files”. Finally, the crowns were cemented to the abutments using glass ionomer cement (Hy-Bond GlasIonomer CX; Shufu Global, Kyoto, Japan) (Fig. 7).

**Fig. 8:**
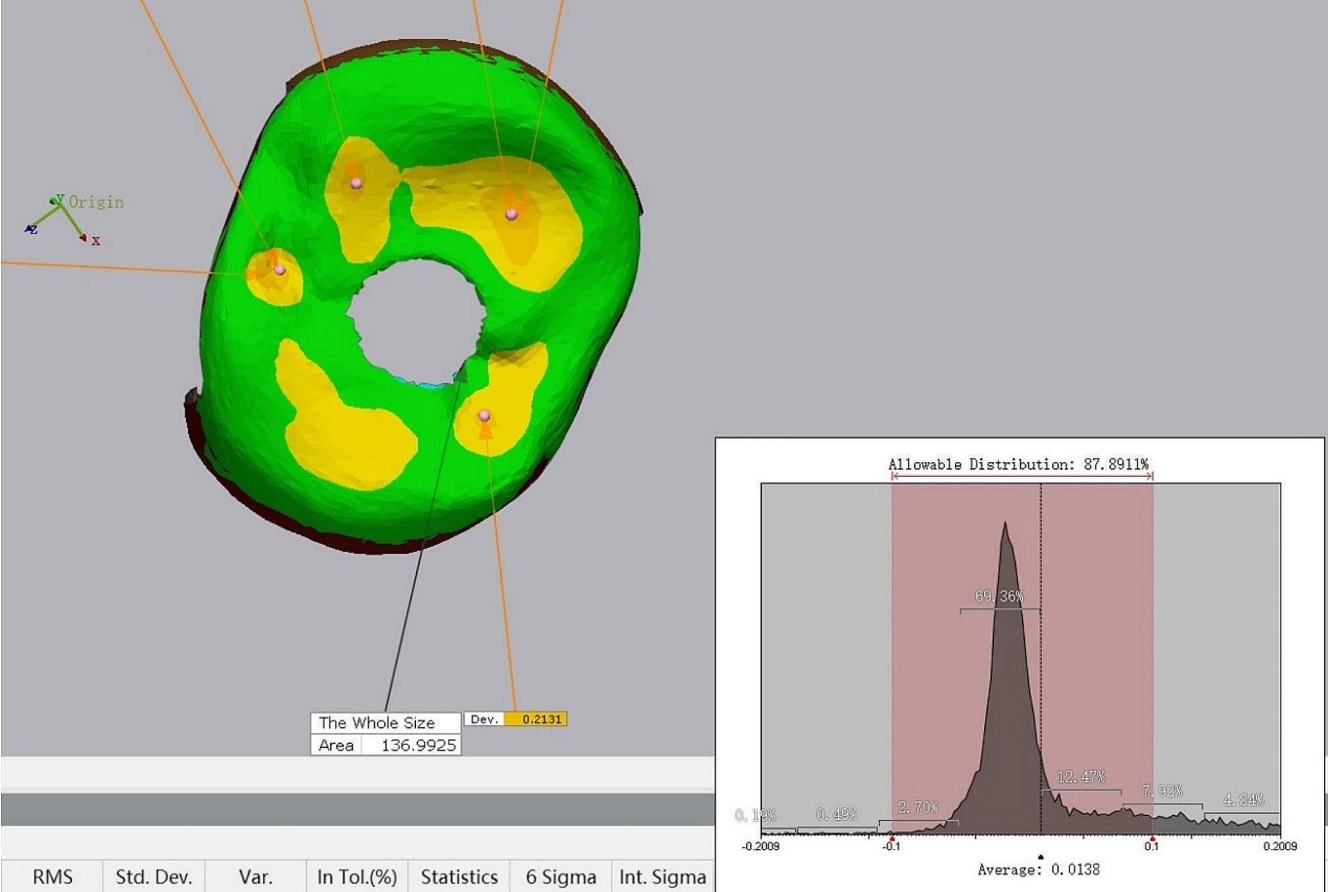
Color coded map of occlusion adjustment. Maximum vertical adjustments extracted from orange area. Allowable distribution on the left corner showed percentage of green area (deviation less than 100 μm)

PRE and POST files were exported to the analysis software (Geomagic Control 2014; Geomagic, NC, USA) for crown adjustment evaluation. POST files were superimposed on the PRE files using an algorithm for best-fit alignment. Mesial and distal interproximal surfaces were then trimmed off and classified as “INTERPROXIMAL” for both PRE and POST files. The remaining files were classified as “OCCLUSION”. Three-dimensional deviation analysis between PRE and POST files was performed for both the INTERPROXIMAL and OCCLUSION files. A color-coded 3D deviation map was generated for each superimposition to facilitate visual analysis. The maximum vertical adjustment, indicated by the deepest color, was extracted from 3D maps for the INTERPROXIMAL and OCCLUSION files. Areas of vertical adjustments less than 100 μm appeared green on the color-coded OCCLUSION map, while the size of the other areas was calculated to determine the extent of occlusal adjustment (Fig. 8).

The duration of clinical and laboratory workflows in minutes was recorded by an independent investigator. The clinical workflow included recording the time required for impression and crown delivery (occlusal and interproximal contact adjustments). The laboratory workflow encompassed the time spent on fabrication, intra-oral scanning, restoration design and manual adjustments and polishing. Waiting time, such as milling and sintering times, was not included in the analysis.

Sample size was calculated based on a previous study using a two-sample t test to exam clinical crown adjustment, with standard deviations of 237 μm in the test group and 485 μm in the control group (mean difference = 248 μm) [5]. With a confidence level of 95%, a significant difference could be elucidated with 8 participants per group. The normality distribution of the data was evaluated using the Shapiro-Wilk test. Where distribution was compatible with normality, the mean, standard deviation and 95% confidence intervals were used. Independent t test, Chi-square test and Fishers’ exact test were used to explore the homogeneity of the demographic data in two groups.

The primary objectives of this study were to assess the effectiveness of an occlusal auxiliary device in improving crown occlusion accuracy, specifically focusing on maximum occlusion adjustment and area of occlusion adjustment, which represent the depth and size of occlusion adjustment, respectively. For repeated observations (mesial crown and distal crown) were available in each patient, a linear mixed model was applied to determine the significant differences of maximum occlusion adjustment and area of occlusion adjustment between the test and control groups.

Furthermore, the secondary outcomes of the study included crown proximal adjustment, as well as the duration of both the clinical and laboratory phases. To compare crown proximal adjustment between the test and control groups, a linear mixed model was utilized. Additionally, an independent-sample t-test was performed to compare the time taken for the clinical and laboratory phases between the two groups. SPSS Statistics software (version 22.0; IBM Corp., Armonk, NY, USA) was used for statistical analyses. A 5% significance level was used for all tests.

## Results

In total, 24 patients (15 females and 9 males) with a mean age of 53.6 years (range: 31–69 years) were included in the study. Table 1 provides a comprehensive overview of the demographic characteristics, ensuring homogeneity in data representation for both the test and control groups. No statistically significant differences were identified between the two groups concerning age, edentulous area classification (free saddle or not), and missing tooth positions (molars or premolars). There were no losses or exclusions after randomization.

**Table 1:** **Demographic characteristics of participants** The age comparison between the two groups employed an independent t-test. Gender and edentulous positions were compared using the Chi-square test, while missing tooth positions were compared using Fisher’s exact test in the two groups

	Test	Control	*p*-value
Participants	12	12	
Age (Years ± SD)	56.2 ± 10.9	50.9 ± 9.6	0.218
Gender			0.673
Female	8 (12)	7 (12)	
Male	4 (12)	5 (12)	
Edentulous position			1.000
Free-end saddle	9 (12)	9 (12)	
Non-free-end saddle	3 (12)	3 (12)	
Missing teeth position			Pairwise comparison
Two molars	6 (12)	8 (12)	
First molar and the second preomolar	5 (12)	2 (12)	0.361
Two premolars	1 (12)	2 (12)	0.500

Table 2 displays the clinical crown occlusion adjustments, including maximum occlusion adjustment, and area of occlusion adjustment in both the test and control groups. A linear mixed model was used to analyze the effect of the two workflows on crown occlusion accuracy and possible influencing factor, crown position (mesial and distal). The group variables (digital and control groups) and crown position were treated as fixed effects and the intercept was included as the random effect. The maximum occlusion deviation was 279.67 ± 112.17 μm and 479.59 ± 203.63 μm in the test and control group, respectively (*p* < 0.001). Additionally, the sizes of occlusion adjustment areas were 12.12 ± 10.51 mm^2^ and 25.12 ± 14.14 mm^2^ in the test and control groups, respectively (*p* = 0.013). Crown positions showed no significant effect on crown occlusion accuracy (*p* = 0.579). The findings indicate that the digital workflow led to significantly greater accuracy of the occlusal surface, as evidenced by the depth and size of the occlusal adjustment. However, crown position didn’t have an impact on the accuracy of crown occlusion.

**Table 2:** Crown occlusion adjustment in test and control groups

		Test	Control	*p*-value
		Mean ± SD (95%CI)	Mean ± SD (95%CI)	
Maximum Occlusion Adjustment (µm)	Mesial Crown	263.74 ± 132.57 (179.51, 347.97)	468.82 ± 208.54 (336.31, 601.32)	
	Distal Crown	295.59 ± 90.45 (238.12, 353.06)	490.36 ± 207.26 (358.67, 622.04)	
	Mean	279.67 ± 112.17 (232.30, 327.03)	479.59 ± 203.63 (393.60, 565.57)	< 0.001
Area of Occlusion Adjustment (mm^2^)	Mesial Crown	10.48 ± 9.13 (4.68, 16.28)	25.16 ± 15.37 (15.41, 34.92)	
	Distal Crown	13.76 ± 11.91 (6.19, 21.89)	25.08 ± 13.50 (16.50, 33.66)	
	Mean	12.12 ± 10.51 (7.68, 16.56)	25.12 ± 14.14 (19.15, 31.09)	0.013

SD: Standard deviation; 95% CI: 95% Confidence interval. A linear mixed model was conducted to evaluate fixed effect of groups and crown positions on maximum occlusion adjustment and area of occlusion adjustment of crowns

The study found that the maximum mesial deviation was 90.24 ± 94.14 μm in the test group and 51.60 ± 55.66 μm in the control group, respectively. Similarly, the maximum distal deviation was 46.38 ± 43.66 μm and 33.54 ± 46.96 μm in the test and control group respectively (Table 3). A linear mixed model was employed to assess the impact of the two workflows on crown proximal accuracy and potential influencing factors, such as crown positions (mesial and distal crown) and proximal sites (mesial and distal surfaces). The analysis revealed that the group variables and crown positions did not have a significant effect on maximum proximal adjustment (*p* = 0.078 and *p* = 0.538 respectively). However, the proximal sites demonstrated a significant effect (*p* = 0.010), indicating that the proximal adjustment varied depending on whether it was the mesial or distal surface. Specifically, the mesial surface necessitated more adjustment compared to the distal surface.

**Table 3:** Crown proximal adjustment in test and control groups

Maximum Proximal Adjustment (µm)	Test		Control	
Mean ± SD (95%CI)	Mesial Crown	Distal Crown	Mesial Crown	Distal Crown
Mesial Surface	81.75 ± 105.63 (14.63, 148.87)	98.73 ± 84.94 (44.76, 152.69)	69.30 ± 66.43 (27.09, 111.51)	44.83 ± 46.45 (15.31, 74.34)
Distal Surface	54.83 ± 46.93 (25.00, 84.64)	37.93 ± 40.35 (12.28,63.57)	33.22 ± 40.04 (7.79, 58.66)	33.85 ± 54.84 (-0.99,68.69)

SD: Standard deviation; 95% CI: 95% Confidence interval. Linear mixed models are conducted to evaluate fixed effect of groups, crown positions and proximal surfaces on maximum proximal adjustment. Mesial surface required statistically more adjustment compared to the distal surface. (*p* = 0.010)

The chair-side times in test and control groups were presented in Table 3. The test group required significantly less time (22.08 ± 3.23 min) compared to the control group (27.75 ± 3.72 min) (*p* = 0.001) in crown delivery. This reduction in time can be attributed to the fact that fewer occlusal adjustments were required in the test group. However, when comparing the time required for impressions recorded using IOS with an auxiliary occlusal device (19.33 ± 4.81 min) to conventional impressions (14.75 ± 2.34 min), the results show that there was a statistically significant difference (*P* = 0.009). Specifically, the use of an auxiliary occlusal device did prolong the duration of impression taking. It is worth noting that despite the differences in chair-side times for the clinical crown adjustment and impressions, the total chair-side times were similar between the test and control groups (*P* = 0.680).

In the dental laboratory, the manufacturing steps and working times for technicians were significantly reduced by the digital workflow. Specifically, the control group required a plaster cast and model scan, which took an average of 33.00 ± 2.26 min, while this step was eliminated entirely in the test group. Furthermore, the use of simultaneous abutment and crown design and milling in the test group eliminated the need for scannings of the abutment model. The CAD times in the test group were then significantly reduced, with an average time of 33.33 ± 3.20 min compared to the control group (53.17 ± 4.04 min). As a result, the total time required in the test group was only 46.08 ± 5.45 min, which is half the time required in the control group (105.92 ± 6.10 min) (*p* < 0.001). The details of the data are shown in Table 4.

**Table 4:** Clinical and laboratory time in minutes in the test and control groups

	Test Mean ± SD (95%CI)	Control Mean ± SD (95%CI )	*p*-value
**Clinical Chairside Time**			
**Impression**	19.33 ± 4.81 (16.28,22.39)	14.75 ± 2.34 (13.26,16.24)	0.009
**Crown Delivery**	22.08 ± 3.23 (20.03,24.14)	27.75 ± 3.72 (25.39,30.11)	0.001
**Total**	41.42 ± 7.04 (36.94,45.89)	42.50 ± 5.57 (38.96,46.04)	0.680
**Laboratory Time**			
**Plaster cast and model scan**	0	33.00 ± 2.26 (31.57,34.43)	0.000
**CAD**	33.33 ± 3.20 (31.30,35.37)	53.17 ± 4.04 (50.60,55.73)	0.000
**Manual adjustment**	12.75 ± 3.96 (10.24,15.26)	19.75 ± 4.25 (17.05,22.45)	0.000
**Total**	46.08 ± 5.45 (42.62,49.55)	105.92 ± 6.10 (102.04,109.79)	0.000

SD: Standard deviation; 95% CI: 95% Confidence interval. An independent-sample t test was conducted to compare the time taken for the clinical and laboratory phases between the test and control groups

## Discussion

The aim of this study was to evaluate the crown accuracy and time efficiency provided by a complete digital workflow for multiple implant-supported single crowns, using an auxiliary occlusal device, compared to a workflow using conventional impression and stone cast-based CAD/CAM crowns. The complete digital workflow on multiple implants with the use of an auxiliary occlusal device demonstrated superior crown accuracy, especially on occlusal surface. Laboratory time was significantly shortened in complete digital workflow, while clinical time showed no difference.

In addressing potential sources of bias in our study, comprehensive measures were implemented to enhance the robustness of our findings. A randomized control study was preferred over a two-way randomized crossover design due to budget constraints, as creating two sets of abutments and zirconia crowns for each patient was financially impractical. Furthermore, implementing two interventions for a single patient would require additional intraoral scanning (IOS) sessions and crown deliveries, posing challenges in terms of time and patient cooperation. Opting for a randomized control group strategically addresses these concerns and mitigates potential biases. The demographic table also confirms the homogeneity of impact factors across the two groups. The dental technician responsible for crown manufacturing and scanning before and after clinical adjustments and researchers who made 3D model alignment and measurements were deliberately blinded to the study design, ensuring an unbiased evaluation of crown adjustments. While the overseeing dentist was aware of the group allocation, adherence to strict criteria for occlusal adjustment aimed to minimize any potential bias in the main outcome.

To fabricated successful prostheses, it is crucial to accurately reproduce the interocclusal relationship and transfer it to the articulator [20, 21]. Accurately transferring the interocclusal relationship to the CAD-CAM software program is essential for providing clinically acceptable restorations with accurate occlusal morphology, reducing the need for chairside occlusal adjustment. However, when multiple teeth are missing, the accuracy of VIR may not meet clinical standard, rendering a complete digital workflow on multiple implants invalid [1, 22, 23]. Previous research [17] explored the use of healing abutments to improve interocclusal relationship accuracy in IOS on stone models, however no clinical study of a complete digital workflow on multiple implant-supported crowns was reported. Therefore, this study represents a significant contribution to the field. The results demonstrate that complete digital workflows reduce the need for occlusal adjustment (in both depth and size) of crowns, as well as laboratory time, compared to conventional workflows. These findings suggest that model-free digital workflows may be suitable for multiple-implant supported restorations, expanding their indications.

In model-free digital workflows, there are generally two approaches to designing and fabricating restorations. The first approach, which is well-established, involves implant-supported screw-retained restorations comprising prefabricated Ti-base abutments and monolithic zirconia crowns. Several studies [5, 24, 25]have reported this full digital approach to be feasible, accurate, and efficient for single implants. However, when it comes to restoring crowns on multiple adjacent implants, particularly when implants are not perfectly parallel, clinicians face challenges in determining the correct insertion path for delivering adjacent screw-retained crowns, especially in the absence of a physical model in the full digital workflow. The second approach involves implant-supported cement-retained restorations, which utilize customized abutments and monolithic zirconia crowns. The use of customized abutments allows for adjustments to the insertion path, resulting in easier and faster delivery of cement-retained crowns. Previous studies [4] have reported and demonstrated the accuracy and efficiency of this approach for single implant-supported crowns. Therefore, customized abutments and cement-retained crowns were chosen to restore on multiple implants in digital and control groups.

The inclusion of a complete digital workflow without auxiliary occlusal devices as an ideal control group was initially considered. However, a preliminary study revealed that crowns produced through this approach sometimes required massive adjustments, while others exhibited infraocclusion and necessitated refabrication in the dental laboratory. Consequently, it was deemed inappropriate to select the complete digital workflow without auxiliary occlusal devices as the control group. Previous studies [4, 25, 26] investigating crown accuracy in digital workflows have utilized the conventional workflow as the control group. This conventional workflow involves conventional impressions, implant-level scanning, abutment CAD/CAM, abutment-level scanning, crown CAD/CAM, and crown delivery. Separate laboratory scans were performed on implant level and abutment level, as it is the most commonly employed method for fabricating cement-retained implant-supported restorations. The mean crown accuracy with the conventional workflow, using the occlusion record recommended in prior studies [27], was found to be 479.59 ± 203.63 μm in this study. This accuracy level is comparable to the crown accuracy observed in conventional workflows for single restorations, which recorded measurements from 330.7 μm to 485 ± 194 μm in previous studies [4, 5, 28].

In this study, proximal adjustment was a secondary outcome measure. It was observed that the mesial surface required more adjustment compared to the distal surface in both the test and control groups. In cases with free-end saddles, no adjustment was deemed necessary on the distal surface of the distal crown, which could be attributed to less need for distal adjustment. Additionally, when the contact between two implant-supported crowns was excessively tight, clinicians might choose to adjust either the distal surface of the mesial crown or the mesial surface of the distal crown based on their personal preference, which could have an impact on the results. However, since the primary focus of this study was the accuracy of occlusion, meticulous clinical instructions regarding proximal adjustment were not established. Overall, proximal adjustments showed no significant difference in two groups, indicating similar accuracy between digital and conventional impressions in terms of the positions of implants and neighboring teeth.

The study compared the clinical chair-side times for digital and conventional impressions and found no significant difference between the test and control groups. However, the time required for digital impressions was approximately 5 min longer than for conventional impressions. This result contradicts previous clinical studies [4, 5, 25, 29] which reported significantly shorter times for intraoral scanning (IOS). One possible explanation for the longer digital impression time could be the use of auxiliary devices, such as installing and uninstalling them, and adjusting their occlusion, which may have contributed to the prolonged process. Despite the longer impression time, the crown delivery process was more time-efficient in the digital workflow than in the conventional workflow, as less occlusion adjustment was needed in the digital group. Overall, the digital workflow did not demonstrate time-efficient superiority over the conventional workflow.

The benefits of the digital workflow for laboratory procedures were obvious; IOS obviates the need for plaster casting and model scanning, while the split-file technique allows designing and milling abutments and crowns in one step, circumventing the need for a second digital model. Several manual fabrication steps were skipped, so the laboratory time was dramatically reduced from 105.92 min in the control group to 46.08 min in the test group. However, it should be reminded that digital workflow requires highly experienced dental technicians because the model-free workflow eliminates the possibility of abutment and crown adjustment on the stone model. The dental technicians should consider abutment marginal depth, path of insertion, proximal contact, and occlusal morphology of restorations in one virtual design. And when the abutments and crowns are milled, only the abutment-crown marginal fit could be adjusted. The inappropriate design on abutment and crown morphology could not be realized until the clinician try them intraorally. The importance of technicians’ experience in CAD/CAM process was mentioned by previous reports [30, 31], and should be highlighted in this study for designing restorations on multiple implants is more complex and challenging.

The advantages of the digital workflow for laboratory procedures were evident in this study. Intraoral scanning (IOS) eliminates the need for plaster casting and model scanning, while the split-file technique enables designing and milling abutments and crowns in one step, thus avoiding the need for a second digital model. These steps reduce several manual fabrication processes, resulting in a significant reduction in laboratory time from 105.92 min in the control group to 46.08 min in the test group.

However, it is worth noting that the digital workflow requires highly experienced dental technicians because the model-free workflow eliminates the possibility of adjusting the abutment and crown on the stone model. Technicians must consider the abutment marginal depth, path of insertion, proximal contact, and occlusal morphology of the restorations in one virtual design. When the abutments and crowns are milled, only the abutment-crown marginal fit can be adjusted. Any inappropriate design on the abutment and crown morphology may not be recognized until the clinician tries them intraorally. The importance of technicians’ experience in the CAD/CAM process has been highlighted in previous reports [30, 31] and this study underscores the complexity and challenges involved in designing restorations on multiple implants.

Several limitations of this study should be acknowledged. Firstly, the sample size was relatively small, which may affect the generalizability of the findings. Secondly, the study didn’t analyze free-end saddle and non-free-end saddle separately, which might affect the result differently, regardless of whether a digital or conventional workflow. Further study should focus on the effect of auxiliary devices in digital workflow for free-end saddle and non-free-end saddle cases individually. Thirdly, while we have shown the validity of the digital workflow for two adjacent implant-supported crowns, further studies are necessary to investigate its applicability for more than two posterior implants.

## Conclusions

This randomized controlled trial (RCT) provides compelling evidence that a digital workflow for multiple implant-supported crowns using an auxiliary device is a feasible and efficient approach. Compared to conventional workflow, the digital workflow required fewer occlusal crown adjustments, and less laboratory time. These findings suggest that the digital workflow has significant advantages for the fabrication of multiple implant-supported crowns. However, the applicability of this digital workflow to more complex cases with extended edentulous spans remains uncertain, and further clinical research is necessary to investigate its feasibility and efficacy.

## Data Availability

The datasets used and/or analysed during the current study available from the corresponding author on reasonable request.
